# Structural characterization and Pickering emulsifying ability of Tartary buckwheat starch prepared by natural deep eutectic solvent method

**DOI:** 10.3389/fnut.2026.1903939

**Published:** 2026-07-07

**Authors:** Qingsheng Zhou, Shijie Zhang, Kitawaki Hidetosh, Xingjun Xie, Zhiqi Zhao, Benguo Liu

**Affiliations:** 1 School of Geography and Tourism, Zhengzhou Normal University, Zhengzhou, China; 2 School of Food Science, Henan Institute of Science and Technology, Xinxiang, China; 3 Faculty of Global and Regional Studies, Toyo University, Tokyo, Japan

**Keywords:** characterization, natural deep eutectic solvents, physicochemical properties, Pickering emulsion, Tartary buckwheat starch

## Abstract

In this study, a betaine–ethylene glycol natural deep eutectic solvent (NADES) system was evaluated as a model extraction medium for preparing Tartary buckwheat starch (TBS). The extraction performance, granule morphology, selected physicochemical properties, and Pickering emulsion-forming behavior of NADES-extracted TBS (NADES-TBS) were compared with those of alkali-extracted TBS (AE-TBS). The highest starch content was obtained at a betaine/ethylene glycol molar ratio of 1:4, water content of 15%, and extraction time of 4 h. Under these conditions, the starch content of NADES-TBS was 87.34 ± 0.81%, which was not significantly different from that of AE-TBS (87.84 ± 0.66%), whereas the apparent amylose content was lower (22.95 ± 0.08% vs. 24.14 ± 0.38%). NADES-TBS showed a slightly larger mean particle size (9.194 μm) than AE-TBS (8.329 μm), while both samples retained the A-type crystalline pattern. The relative crystallinity changed only slightly from 30.39 to 31.11%. Compared with AE-TBS, NADES-TBS showed a higher pasting temperature, lower peak viscosity, and lower setback viscosity. In an MCT–water model system, NADES-TBS showed lower oil–water interfacial tension and a contact angle closer to 90° than AE-TBS. Pickering emulsions stabilized by NADES-TBS showed smaller droplet sizes than those stabilized by AE-TBS under the tested short-term conditions. These results indicate that the selected NADES extraction process was associated with changes in granule morphology and selected physicochemical/interfacial properties of TBS. However, because residual ethylene glycol, molecular-level structure, and long-term emulsion stability were not fully evaluated, food-related application and mechanistic conclusions require further verification.

## 1 Introduction

As the basic raw material of many industries such as food, medicine and chemical industry, the preparation method of grain starch with high efficiency, high purity and low damage is an important research direction of modern grain processing technology. The industrial preparation of traditional cereal starch (such as corn, wheat and rice starch) is mainly based on wet grinding process, and its core is to realize component separation through soaking, crushing, separation and refining (1). Specifically, for corn starch, sulfurous acid soaking method is widely used, which uses the antiseptic, dissolution-promoting and weak bleaching effects of sulfur dioxide to soften seeds and initially dissolve soluble proteins and pigments; Then, the primary separation was realized by multi-stage grinding, cyclone separation and centrifugation, using the specific gravity difference between starch and protein. Wheat starch is often extracted by gluten washing method. Wet gluten and starch milk are obtained by kneading and washing, and then starch is obtained by centrifugation or precipitation. Although these traditional methods are mature in technology, there are widespread problems such as complex process, huge water consumption and high wastewater pollution load (2, 3). In addition, the traditional methods have limited removal efficiency of endogenous proteins and deep pigments closely combined with starch particles, and it is difficult to meet the market demand of high-purity and high-whiteness special starch (4).

Natural deep eutectic solvents (NADESs), as a green solvent system formed by the primary metabolites of plants (such as choline, organic acids, sugars and alcohols) through hydrogen bonding, has been used to extract polyphenols and proteins because of its designable physicochemical properties, low toxicity, biodegradability and high solubility (5). The solvent can simultaneously remove pigments and proteins from grains, which provides a revolutionary solution for preparing high-purity starch. The potential advantages of preparing starch by NADES are mainly reflected in three aspects: first, high efficiency and selectivity. By reasonably designing the components of NADESs (such as choline chloride-lactic acid, betaine-glucose, etc.), its unique hydrogen bond network can effectively dissolve and extract a variety of pigments (such as carotenoids and polyphenol pigments) and denature or dissolve protein, and at the same time, it has little damage to the structure and functional characteristics of starch granules, so as to realize deep decoloration and deproteinization in one step or simplified process, and obtain starch products with higher whiteness and purity (6). The second is green and sustainability. NADES raw materials are derived from biomass, and the production process is environment-friendly, and the solvent can be efficiently recovered and recycled by vacuum distillation, membrane separation and other methods, thus greatly reducing or even avoiding the use of toxic chemicals and the discharge of high-salt organic wastewater from the source, which conforms to the principles of green chemistry and clean production (7). The third is the improvement of product quality. Based on the treatment of NADES, the functional characteristics of the obtained starch may also change, expanding its application field (8). Pickering emulsions are emulsions stabilized by solid particles rather than conventional molecular surfactants. The irreversible or strong adsorption of particles at the oil–water interface can improve emulsion stability and reduce the need for synthetic surfactants. Starch particles are attractive Pickering stabilizers because they are natural, edible, biodegradable, abundant, and inexpensive. However, native starch often shows limited interfacial activity due to its strong hydrophilicity (9). Therefore, extraction or modification strategies that alter starch morphology, wettability, or interfacial behavior may improve its Pickering emulsifying ability. Based on this consideration, the present study investigated whether NADES extraction could improve the interfacial and Pickering emulsifying properties of Tartary buckwheat starch.

Tartary buckwheat is an annual food crop of Polygonaceae, which originated in Himalayan and southwest China region. It is regarded as an important medicine and food homologous crop because of its poor tolerance, cold tolerance and short growth period. Tartary buckwheat seeds are rich in protein, dietary fiber, flavonoids (such as rutin) and various minerals (10, 11), and its starch, as the main component of Tartary buckwheat (about 60–70% of the dry weight of the seeds), belongs to compound starch particles, and its particle size is usually small and polygonal; Structurally, it shows high amylose content and contains unique resistant starch components, which makes it show high gel temperature, slow digestion rate and low glycemic index. These physical and chemical characteristics make Tartary buckwheat starch show important application potential in functional food development, dietary intervention for chronic diseases and preparation of biodegradable materials. Previous studies have shown that deep eutectic solvents can interact with starch and influence starch processing behavior (12). However, limited information is available on the preparation of Tartary buckwheat starch using NADES systems and on the resulting Pickering emulsion-forming behavior. It should be noted that the safety and applicability of NADES-treated starch depend strongly on the solvent components and residual solvent levels. Therefore, the present work should be regarded as a model-system investigation rather than a direct food-application validation. In this study, a betaine–ethylene glycol NADES system was used to prepare Tartary buckwheat starch (NADES-TBS), and its properties were compared with those of starch prepared by conventional alkaline extraction (AE-TBS). The objective was to evaluate whether this extraction workflow is associated with changes in granule morphology, selected physicochemical properties, wettability, and short-term Pickering emulsion-forming behavior.

## 2 Materials and methods

### 2.1 Materials and chemicals

Tartary buckwheat flour was from our previous report (13). Betaine and ethylene glycol and medium-chain triglyceride (MCT) were purchased from Yuanye Biotechnology Co., Ltd. (Shanghai, China). All other chemicals were of analytical grade.

### 2.2 Preparation of NADES

Betaine and ethylene glycol were mixed in a certain betaine/ethylene glycol molar ratio (1:2, 1:3, 1:4, 1:5, 1:6), and a certain amount of water was added until the water content of the system reached the designated value (0, 5, 10, 15, 20%). The mixture was heated at 80 °C until a clear liquid formed. The resulting liquid was sealed and stored at 4 °C for subsequent experiments.

### 2.3 Preparation of Tartary buckwheat starch by NADES method

The preparation procedure was adapted from previous studies on starch extraction and deep eutectic solvent treatment (8, 13). The Tartary buckwheat flour was soaked and stirred with 15 volumes of n-hexane for 6 h, the filtrate was discarded. This operation was repeated 3 times to remove lipids. The obtained defatted Tartary buckwheat flour was used for subsequent starch preparation. The defatted Tartary buckwheat flour was soaked in 10 volumes of specific NADES solutions with varying water contents (0, 5, 10, 15, 20%) and betaine/ethylene glycol molar ratios (1:2, 1:3, 1:4, 1:5, 1:6), and stirred continuously for a set period (1 h, 2 h, 3 h, 4 h, 5 h). The mixture was centrifuged at 4000 rpm for 10 min, the filtrate was removed, and the precipitate was collected, washed three times with water, dried at 40 °C, and crushed through a 100-mesh sieve to obtain NADES-extracted Tartary buckwheat starch (NADES-TBS).

### 2.4 Preparation of Tartary buckwheat starch by alkaline method

The alkaline extraction procedure was performed according to previously reported methods with slight modifications (4). The defatted Tartary buckwheat powder was mixed with 10 times the volume of 70% ethanol, stirred at room temperature for 30 min, centrifuged at 4000 rpm for 15 min. The supernatant was discarded, and this process was repeated 3 times to remove pigments and phenolic compounds. The obtained Tartary buckwheat flour was soaked in 10 volumes of NaOH solution (pH 10.0), stirred continuously for 6 h, and then centrifuged at 4000 rpm for 15 min. The supernatant was discarded, and the precipitate was collected, adjusted to pH 7.0, and washed repeatedly with water three times. It was then dried at 40 °C, ground, and sieved through a 100-mesh sieve to obtain alkali-extracted Tartary buckwheat starch (AE-TBS).

### 2.5 Measurement of starch content and amylose content

The starch content was measured using a polarimetric method (14). A 0.75 g starch sample was mixed with 50 mL of 1% HCl solution, heated in a boiling water bath for 15 min, and then rapidly cooled to room temperature. To the cooled starch solution, 1 mL of 30% ZnSO_4_ solution and 1 mL of 15% K_4_[Fe(CN)_6_] solution were added sequentially‌. The volume of the mixture was then adjusted to 100 mL. Its optical rotation was read on an automatic polarimeter, and the starch content was calculated accordingly.

The amylose content was measured based on a colorimetric method (15). The 2.5 mL of different standard solutions containing 1 mg/mL amylose and amylopectin in varying ratios were mixed with 20 mL of distilled water. The pH of the solution was adjusted to 3.0, followed by the addition of 0.5 mL of iodine reagent, and the volume was made up to 50 mL. After standing for 10 min, its absorbance at 620 nm was read. A standard curve was plotted with the amylose/amylopectin ratio as x-axis and the absorbance as y-axis. A sample (50 mg) was mixed with 10 mL of 0.5 mol/L NaOH solution, then shaken at 95 °C for 15 min. The volume was adjusted to 50 mL with distilled water. The 2.5 mL of this sample solution was taken, color-developed following the same procedure described above, and its absorbance at 620 nm was measured. The amylose content in the sample was determined based on the standard curve.

### 2.6 Starch granule morphology and particle size determination

The samples were fixed on a test table with double-sided tape and sprayed with gold, and their morphologies were analyzed using an FEI Quanta 200 environmental scanning electron microscope (Hillsboro, OR, United States) at 2000 times magnification. Subsequently, 0.1% of the sample solution was dropped on a slide and placed under a polarizing microscope to observe the birefringence of the starch particles under polarizing light (16). Finally, the size distribution of the samples was evaluated by using a BETTERBT-9300H laser particle size analyzer (Dandong, China).

### 2.7 X-ray diffraction measurement

The X-ray diffraction (XRD) patterns of the samples in the 2θ range of 10–70° were recorded by a D8 Advance X-ray diffractometer (Bruker, Germany) at a scanning speed of 8°/min and a step size of 0.04° (Livermore, CA, United States) (17). The relative crystallinity was calculated using Jade software by separating the crystalline diffraction peak area from the amorphous background area. The relative crystallinity, RC, was calculated according to the following equation:


RC(%)=Ac/(Ac+Aa)×100

Where Ac is the integrated area of crystalline peaks and Aa is the area of the amorphous region under the diffraction curve.

### 2.8 Determination of starch pasting properties

A 3.0 g starch sample and 25 mL of distilled water was mixed in an RVA 4500 rapid visco analyzer. The mixture was held at 50 °C for 1 min, heated to 95 °C within 3.5 min and maintained at this temperature for 3 min, then cooled to 50 °C within 3.5 min and held at 50 °C for 2 min. The viscosity changes during this process were recorded (18).

### 2.9 Determination of starch gel strength

Samples of 3.2, 4.0, and 4.8 g of starch were placed in 50 mL beakers, and distilled water was added to bring the total mass to 40.0 g. The mixtures were heated in a 95 °C water bath with stirring for 30 min until the starch was completely gelatinized. After removal, they were cooled to room temperature and then allowed to stand in a refrigerator at 4 °C for 16 h. The cylindrical sample with a circular bottom diameter of 4 cm and a height of 3 cm was used for gel strength determination. The measurement was performed on a TA-XT plus texture analyzer (Stable Micro Systems, United Kingdom) with a P/0.5 probe, where the trigger force was 2.0 g, the compression distance was set to 10 mm, and the pre-test, test, and post-test speeds were 1.5, 1.0, and 1.0 mm/s. The force received by the probe when it entered 4 mm into the starch gel was recorded as the gel strength (19).

### 2.10 Interfacial tension and contact angle measurement

The interfacial tension and contact angle of the samples were measured using a Theta Lite optical contact angle meter equipped with One Attension software (Biolin Scientific, Stockholm, Sweden) based on the Young-Laplace equation (20). For interfacial tension measurement, a 20 μL droplet of saturated sample solution was dispensed from a pipette tip immersed in a quartz container filled with MCT. The droplet formation process was recorded for 10 s at a frame rate of 1 frame/s. For contact angle measurements, starch powder (approximately 0.5 g) was compressed using a manual hydraulic press under a pressure of approximately 10 MPa for 1 min to obtain a compact disk with a diameter of 2 cm and a thickness of about 2 mm. The disk was carefully placed in a quartz container filled with MCT, and a 6 μL water droplet was deposited on its surface for contact angle determination. The recording time was 10 s, with a frequency of 1 frame/s, and the corresponding contact angle was calculated using OneAttension software (Biolin Scientific, Stockholm, Sweden). Data analysis and curve fitting were performed using OneAttension software.

### 2.11 Preparation of Pickering emulsions

Appropriate amounts of NADES-TBS and AE-TBS starch samples were added to water as the aqueous phase, with MCT used as the oil phase. The two phases were mixed at a predetermined oil phase volume fraction and homogenized at 15,000 rpm for 3 min to prepare Pickering emulsions with starch concentrations (*c*) of 2–4% (w/v) and oil phase volume fractions (*φ*) of 40–80% (v/v). Emulsion stability was observed after 24 h (21). Emulsion appearance, droplet size, and stability were evaluated immediately after preparation and after storage for 1 days at 4 °C. Before measurement, emulsions were gently equilibrated to room temperature. The storage stability was evaluated by observing phase separation, emulsion appearance, and droplet size changes during storage.

### 2.12 Emulsion microstructure and droplet size measurement

Pickering emulsions were prepared using NADES-TBS and AE-TBS as emulsifiers at starch concentrations (*c*) of 3.5 and 4.0%, and an oil phase volume fraction (*φ*) of 60%. The morphological appearance was observed using a microscope. Droplet size distribution was measured using a laser particle size analyzer. Before measurement, freshly prepared emulsions were diluted with distilled water (1:100, v/v) under gentle stirring to avoid multiple scattering and droplet coalescence. The refractive indices of the oil and aqueous phases were set according to the instrument requirements, and each sample was measured in triplicate. The average volume-weighted droplet diameter was recorded (22).

### 2.13 Measurement of emulsion microrheological properties

Pickering emulsions were prepared using NADES-TBS and AE-TBS as emulsifiers at an oil phase volume fraction (*φ*) of 60% and starch concentrations (*c*) of 3.5 and 4.0%. Their microrheological behavior was measured using a Rheolaser LAB6 microrheometer (Formulation, France). A volume of 20 mL of freshly prepared emulsion was placed into a 25 mL microrheological test bottle and loaded into the instrument’s measurement chamber. The sample was continuously monitored at 25 °C for 4 h using a CCD detector. Data were recorded and processed using Rheosoft Master 1.4 software to obtain the elasticity index (EI) and macroscopic viscosity index (MVI) (23).

### 2.14 Statistical analysis

**Figure 1:**
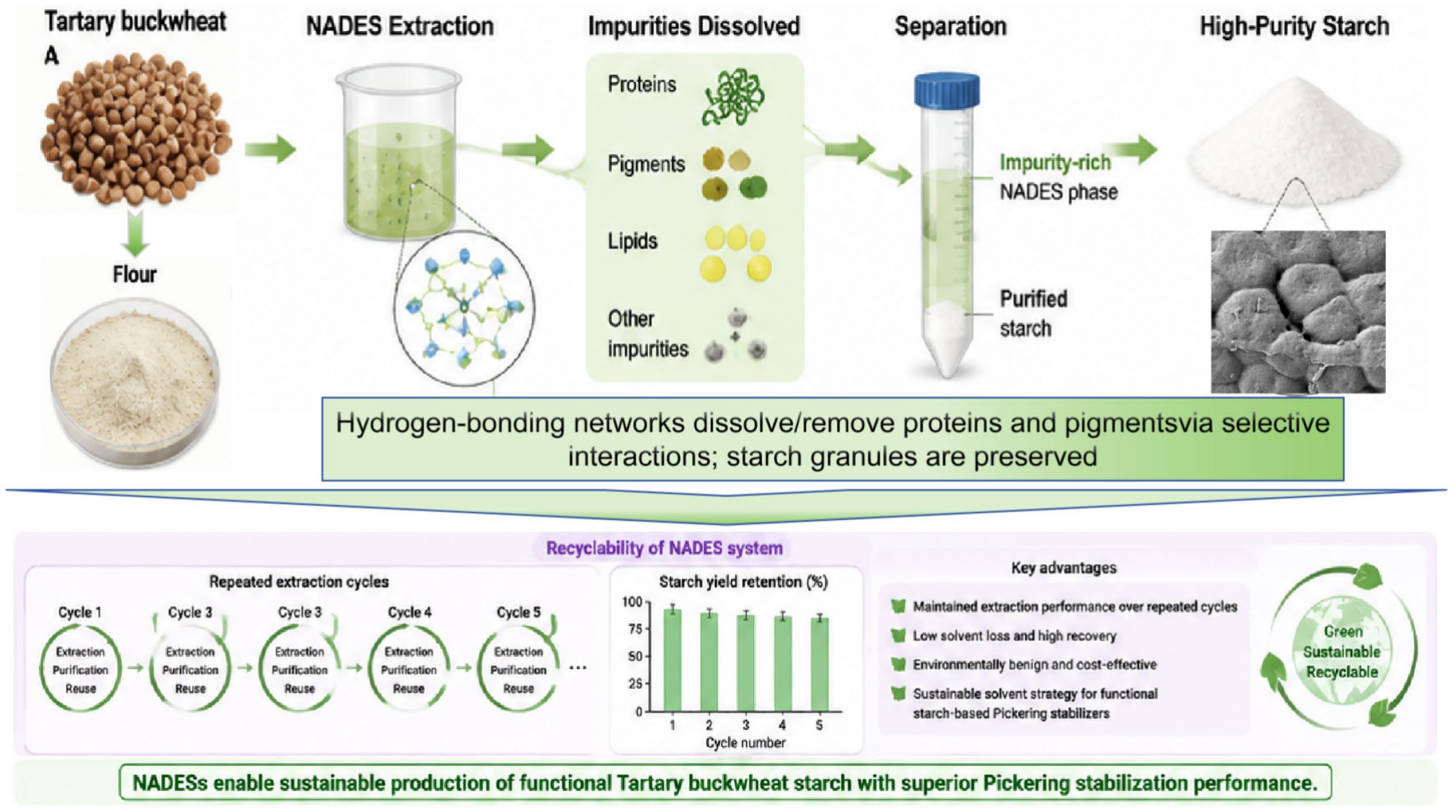
Schematic illustration of NADES-based extraction of Tartary buckwheat starch and its sustainable production mechanism.

Experimental results are expressed as mean ± standard deviation (*n* = 3). Data were processed using SPSS 18.0 software, and one-way analysis of variance (ANOVA) was performed using Duncan’s test. Differences were considered statistically significant at *p* ≤ 0.05 (Figure 1).

## 3 Results and discussion

### 3.1 Preparation of NADES-TBS

**Figure 2:**
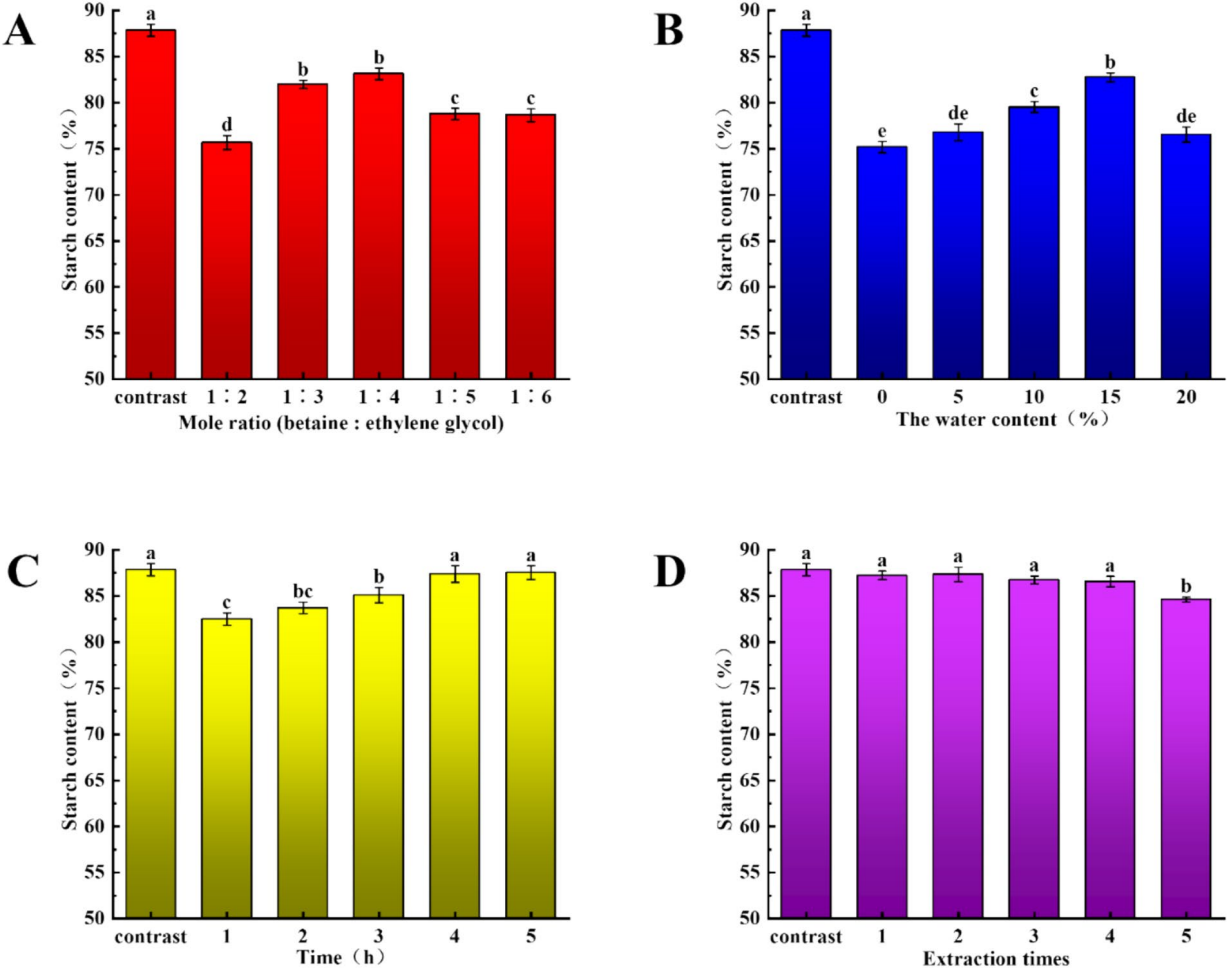
Effects of betaine/ethylene glycol molar ratio **(A)**, water content **(B)**, extraction time **(C)**, and NADES reuse cycles **(D)** on starch content. “control” refers to AE-TBS, namely Tartary buckwheat starch prepared by the conventional alkaline extraction method and used as the reference sample. Different superscript letters indicate statistically significant differences, *p* ≤ 0.05.

NADES is formed by mixing a hydrogen bond donor and a hydrogen bond acceptor in a specific molar ratio. Its composition not only influences its physicochemical properties (such as density, viscosity, and polarity) but also affects its solubility and capacity to dissolve target components (24). As shown in Figure 2A, the highest starch content was achieved when the betaine/ethylene glycol molar ratio was 1:4. This is because the molar ratio between components determines the system’s stability, and the number of hydrogen bond donors and acceptors, spatial structure, and bond positions significantly influence NADES formation (25). The physicochemical properties of NADES are strongly dependent on the number, type, and spatial arrangement of hydrogen-bonding sites in their components. Hydroxyl- and carboxyl-containing compounds can contribute to extensive hydrogen-bond networks, which stabilize the eutectic liquid phase and alter its polarity and viscosity (26). However, excessive hydrogen-bond networking can increase viscosity and reduce mass transfer efficiency. Therefore, the optimal betaine/ethylene glycol molar ratio of 1:4 observed in this study may reflect a balance between solvent stability and extraction efficiency. As shown in Figure 2B, the highest starch content was observed at 15% water content. Adding an appropriate amount of water during NADES preparation or prior to extraction reduces system viscosity, thereby improving impurity removal efficiency. Moreover, water alters system polarity, making it more compatible with the target compound. However, excessive water weakens the hydrogen bonding interactions within NADES, reducing impurity removal efficiency and consequently lowering starch yield. In Figure 2C, starch content reached its maximum after 4 h of extraction, with no significant difference between 4 h and 5 h, indicating that extraction efficiency plateaus beyond 4 h. Therefore, the optimal extraction conditions are a betaine/ethylene glycol molar ratio of 1:4, 15% water content, and an extraction time of 4 h. Furthermore, as shown in Figure 2D, starch content did not significantly decrease after four consecutive extractions using the same NADES, indicating its potential for repeated use.

### 3.2 Comparison of starch composition

Table 1 shows a comparison of the composition between NADES-TBS and AE-TBS‌, obtained under conditions of 15% water content in the system, a betaine/ethylene glycol molar ratio of 1:4, and an extraction time of 4 h, with AE-TBS prepared by the conventional alkaline method. The starch content of NADES-TBS was 87.34 ± 0.81%, which is close to that of AE-TBS (87.84 ± 0.66%), with no significant difference. However, the amylose content of NADES-TBS was 22.95 ± 0.08%, significantly lower than that of AE-TBS (24.14 ± 0.38%). This difference may be related to extraction-associated changes in iodine-binding accessibility, partial leaching of starch fractions during washing, or differences between the NADES and alkaline extraction workflows. However, because molecular-level analyses such as FTIR, SEC/GPC, and molecular weight distribution measurements were not performed, the present data do not allow us to determine whether starch molecular degradation occurred. Therefore, the amylose result should be interpreted as a change in apparent amylose content rather than direct evidence of molecular degradation or molecular structural modification (8). Further characterization, particularly FTIR analysis, is required to evaluate possible changes in short-range molecular order and hydrogen-bond interactions in NADES-TBS.

**Table 1:** Starch content, moisture content, amylose content, and amylopectin content of NADES-TBS and AE-TBS.

Content (%)	NADES-TBS	AE-TBS
Starch content (%)	87.34 ± 0.81^a^	87.84 ± 0.66^a^
Moisture content (%)	8.87 ± 0.16^a^	7.94 ± 0.02^b^
Amylose content (%)	22.95 ± 0.08^b^	24.14 ± 0.38^a^
Amylopectin content (%)	77.05 ± 0.08^a^	75.86 ± 0.38^b^

Values are expressed as mean ± standard deviation (n = 3). Different superscript letters within the same row indicate statistically significant differences between samples (*p* ≤ 0.05).

### 3.3 Microscopic observation of starch

**Figure 3:**
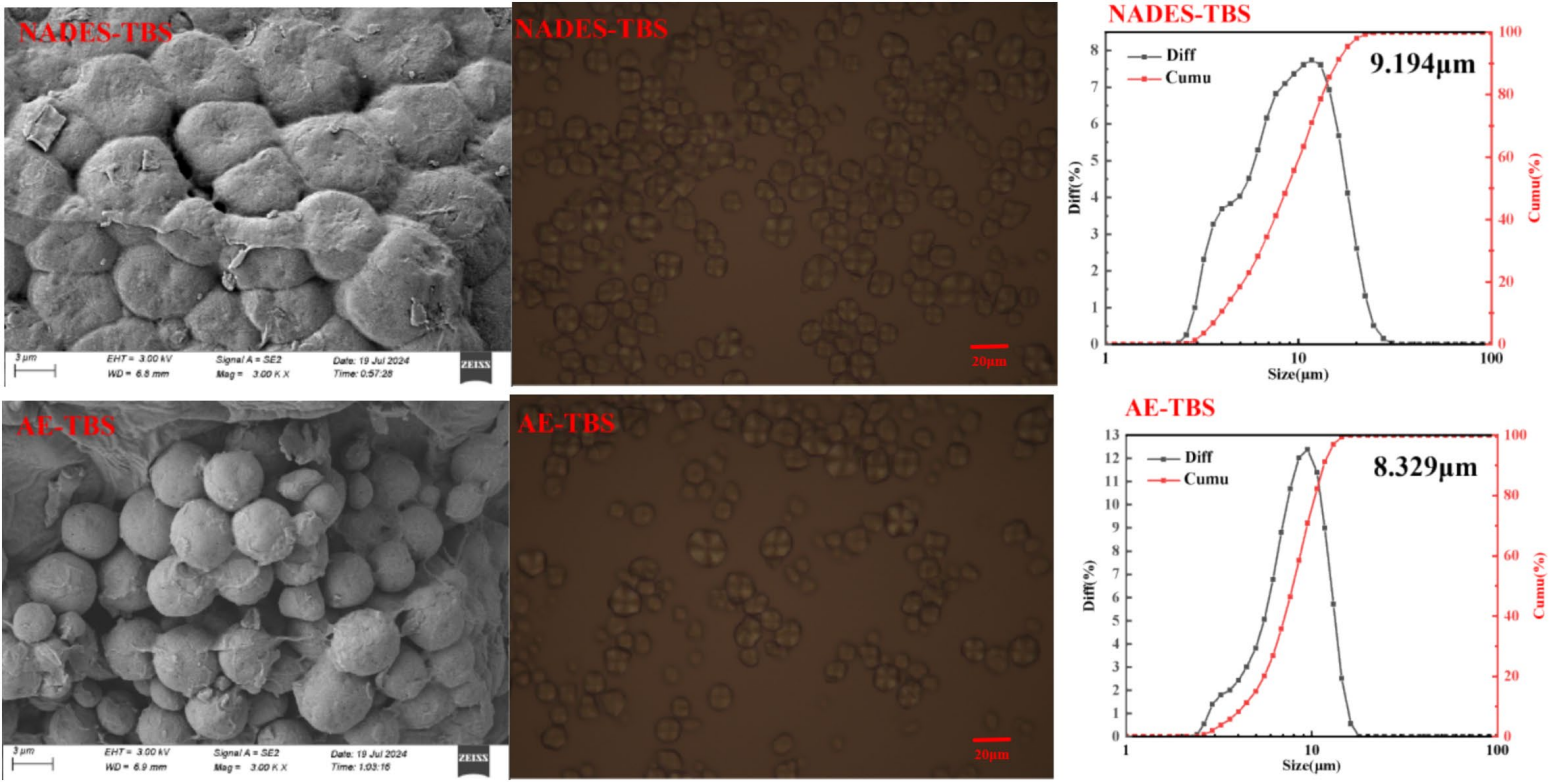
SEM images, polarized light microscopy images, and particle size distributions of NADES-TBS and AE-TBS. SEM images show the surface morphology of starch granules, while polarized light microscopy images show the birefringence patterns of starch granules. The right panel shows the particle size distribution of the starch samples. Scale bars are shown in the corresponding images. Different superscript letters in the same column indicate statistically significant differences, *p* ≤ 0.05.

The appearance and size of starch granules are significantly influenced by their source and extraction method‌ (27). As shown in Figure 3, scanning electron microscopy results reveal that AE-TBS starch granules are smaller, uniformly distributed, and mostly spherical in shape, whereas NADES-TBS granules exhibit a more regular polygonal or nearly circular structure, with noticeable adhesion and aggregation among granules. This may be attributed to the inherent viscosity of NADES, leading to microscopic aggregation. Such aggregation could affect the arrangement of particles at the oil–water interface during subsequent emulsion formation. In Figure 3, both NADES-TBS and AE-TBS granules display birefringence under polarized light microscopy, showing a Maltese cross pattern, indicating that NADES does not disrupt the spherulitic structure of starch. Particle size analysis in Figure 3 shows that the average diameter of NADES-TBS (9.194 μm) is significantly larger than that of AE-TBS (8.329 μm), consistent with the SEM observations, suggesting that physical aggregation of starch granules is more likely during NADES extraction. In contrast, the strong alkaline environment in alkaline extraction may erode the granule surface, weakening inter-particle interactions and promoting the formation of smaller, monodispersed granules during drying and dispersion. The aggregation observed in NADES-TBS may be related to the relatively high viscosity of the NADES medium and the subsequent drying process. In Pickering emulsions, moderate aggregation may be beneficial because aggregated particles can adsorb at the oil–water interface and form a thicker and more compact particulate barrier around droplets. Such a structure may enhance steric hindrance and reduce droplet coalescence. However, excessive aggregation could decrease the effective number of particles available for interfacial coverage. Therefore, the moderate aggregation observed in NADES-TBS may contribute positively to its improved Pickering emulsifying performance (28). Therefore, the increased particle size should be regarded as a secondary but potentially beneficial factor, whereas interfacial wettability and interfacial tension are likely more directly related to the improved Pickering emulsifying ability of NADES-TBS. Zhang et al. also found that different extraction methods alter granule viscosity, promoting aggregation. In the emulsion experiments, interfacial tension and contact angle provide more direct evidence for the different Pickering emulsion-forming behaviors of NADES-TBS and AE-TBS, whereas granule aggregation may be a secondary contributing factor that requires further verification (29).

### 3.4 Crystal structure of starch

**Figure 4:**
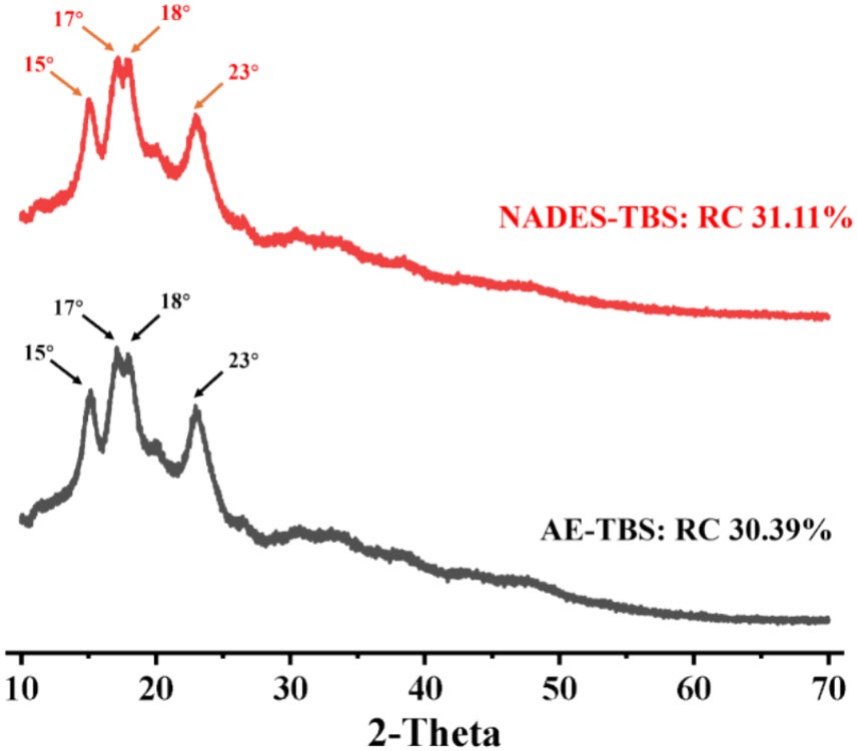
XRD pattern of NADES-TBS and AE-TBS.

Starch is a natural semi-crystalline polymer composed of crystalline regions, amorphous regions, and submicrocrystalline structures. The crystalline characteristics of starch and its derivatives directly influence the functional properties of starch-based products (30). The crystalline features of starch granules are affected by factors such as amylose content and the degree of polymerization of starch molecules, which can be reflected in X-ray diffraction (XRD) patterns. Figure 4 shows the XRD patterns of NADES-TBS and AE-TBS, where sharp peaks indicate the crystalline regions. Both starches exhibit strong diffraction peaks at 15°, 17°, 18°, and 23°, displaying the typical A-type crystalline pattern, indicating that NADES treatment does not alter the crystalline form of Tartary buckwheat starch (31). Compared with AE-TBS, NADES-TBS showed only a slight increase in relative crystallinity, from 30.39 to 31.11%. Therefore, this small difference should be interpreted cautiously. The XRD results mainly indicate that the NADES extraction process did not change the A-type crystalline pattern of Tartary buckwheat starch. The slight increase in relative crystallinity may be related to the removal of amorphous impurities or minor rearrangement of ordered regions, but it is unlikely to be the sole factor responsible for the differences in functional and Pickering emulsifying properties (32).

### 3.5 Analysis of pasting properties

The gelatinization process of starch refers to a series of physicochemical changes that occur when starch granules are heated in the presence of water, including granule hydration and swelling, gradual disintegration of crystalline structure, disappearance of birefringence, and partial dissolution of starch molecules. During this process, the original ordered structure within starch granules is disrupted, and this structural breakdown is typically irreversible. Starch gelatinization behavior significantly influences the textural properties, viscosity characteristics, and water-holding capacity of starch-based food systems. Peak viscosity reflects the maximum viscosity reached during granule swelling and complete gelatinization, and is closely related to factors such as starch granule size, the ratio of amylose to amylopectin, and the chain length distribution of amylopectin branches (33). Breakdown value and setback value are commonly used to evaluate the structural stability of starch during heating and cooling processes. Lower breakdown and setback values generally indicate better heat and shear resistance of starch granules, as well as reduced tendency for retrogradation during cooling, implying superior cold paste stability. Table 2 presents a comparison of the pasting properties between NADES-TBS and AE-TBS. The gelatinization temperature of NADES-TBS is significantly higher than that of AE-TBS, indicating that NADES-TBS granules possess a more stable structure and require a higher temperature to initiate gelatinization. Furthermore, the peak viscosity, trough viscosity, breakdown, final viscosity, and setback of NADES-TBS are all lower than those of AE-TBS. This is attributed to the larger granule size of NADES-TBS, which results in greater structural integrity during gelatinization, making it less prone to rupture. Consequently, NADES-TBS exhibited lower peak and setback viscosities. The lower setback value indicates a weaker tendency of starch chains, especially amylose chains, to reassociate during cooling, suggesting reduced retrogradation tendency and better cold-paste stability. From an application perspective, this property may be advantageous in starch-based sauces, fillings, cold pastes, instant pudding-like products, and emulsion gels, where excessive retrogradation can cause hardening, syneresis, and undesirable texture changes during storage (33). The markedly lower setback viscosity of NADES-TBS suggests a reduced tendency for starch chain reassociation during cooling, which may be advantageous in products requiring better cold-paste stability and slower retrogradation. Such properties may be useful in starch-based sauces, fillings, instant pastes, cold-stored semi-solid foods, or emulsion gels where excessive retrogradation can lead to syneresis, hardening, or texture deterioration during storage.

**Table 2:** Pasting properties of NADES-TBS and AE-TBS.

Detection indicators	NADES-TBS	AE-TBS
Pasting temperature (°C)	77.25 ± 0.75^a^	74.73 ± 0.80^b^
Peak Time (min)	6.42 ± 0.22^b^	6.54 ± 0.12^a^
Peak viscosity (mPa∙s)	2,454 ± 144^b^	3,873 ± 225^a^
Trough viscosity (mPa∙s)	2,353 ± 140^b^	3,675 ± 251^a^
Cold viscosity (mPa∙s)	2,946 ± 149^b^	6,021 ± 366^a^
Breakdown viscosity (mPa∙s)	101 ± 11^b^	197 ± 38^a^
Setback viscosity (mPa∙s)	593 ± 9^b^	2,346 ± 173^a^

Values are expressed as mean ± standard deviation (*n* = 3). Different superscript letters within the same row indicate statistically significant differences between samples (*p* ≤ 0.05).

### 3.6 Gel strength of starch

**Figure 5:**
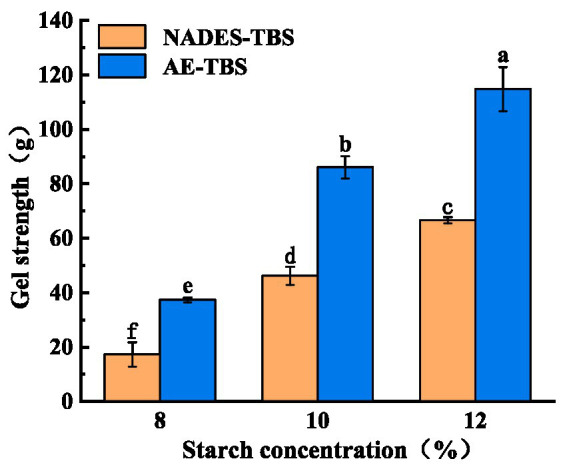
Gel strength values of NADES-TBS and AE-TBS at starch concentrations of 8, 10, and 12%. Different superscript letters indicate statistically significant differences, *p* ≤ 0.05.

The principle of starch gel strength determination is based on the evaluation of the gel structure formed during heating and cooling processes and its mechanical properties. By measuring the degree of deformation of the gel under applied force, its strength, elasticity, and stability can be assessed. The greater the number and density of association points between starch molecules in the gel, the higher the gel strength (34). This study compared the gel strength values of NADES-TBS and AE-TBS at concentrations of 8, 10, and 12% (Figure 5). At each concentration, the gel strength of NADES-TBS was lower than that of AE-TBS, primarily due to its lower tendency for retrogradation and reduced amylose content compared to AE-TBS. As a result, the internal structure of NADES-TBS gel is relatively loose, leading to lower gel hardness. The differences in gel behavior and particle interaction may be related to changes in hydrogen bonding, molecular reassociation, and particle-particle interactions. Starch molecules can form hydrogen bonds through hydroxyl groups, and the NADES extraction environment may affect the accessibility of these groups or the association among starch chains (35). Reduced apparent amylose content and altered granule aggregation may weaken the formation of a continuous gel network, leading to lower gel strength in NADES-TBS. In addition, attraction-repulsion balance among starch particles may influence their dispersion and interfacial adsorption behavior in emulsion systems.

### 3.7 Wettability of starch

**Figure 6:**
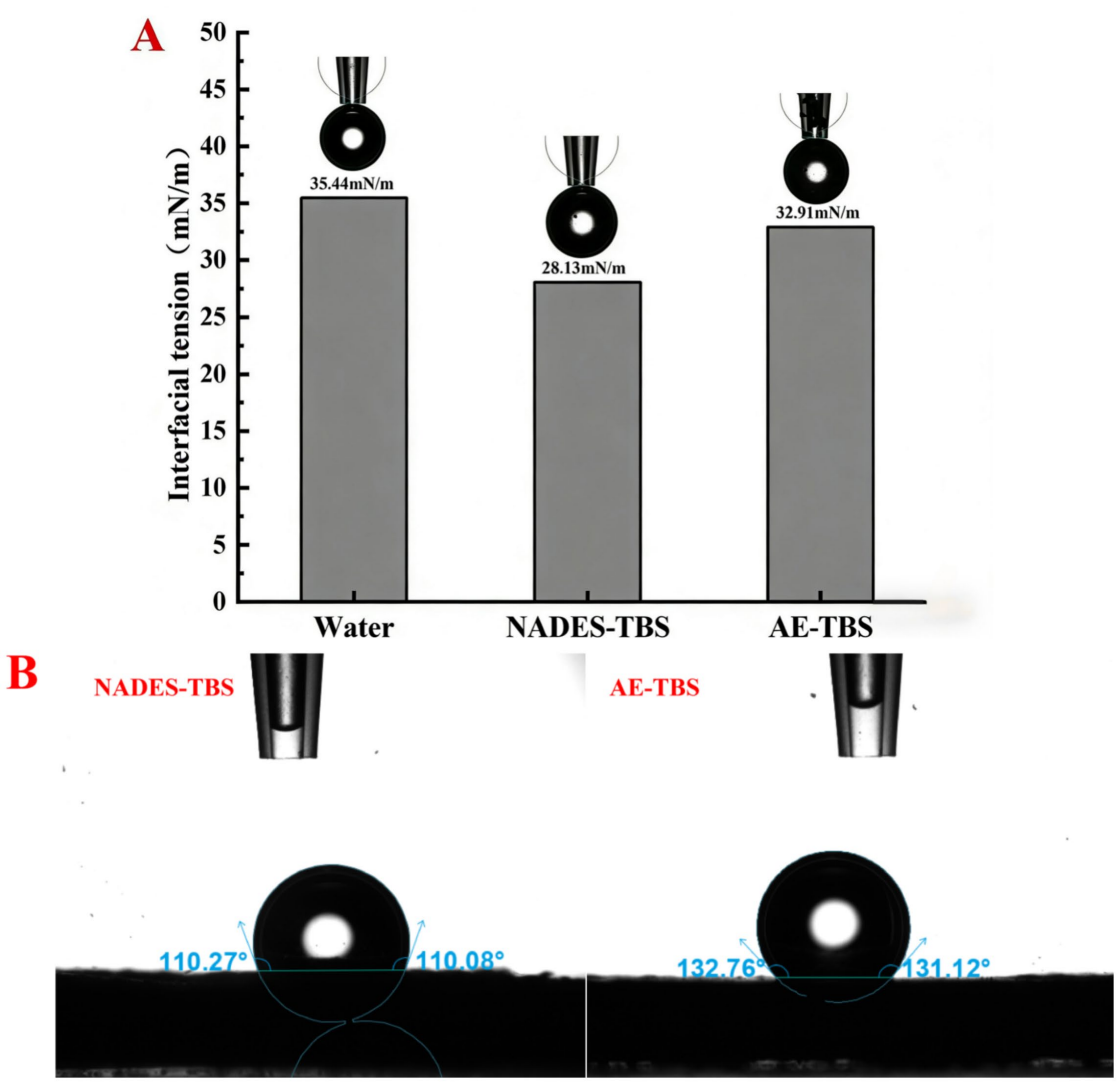
Oil–water interfacial tension in the O/W, oil-in-water, model system **(A)** and three-phase contact angle **(B)** of NADES-TBS and AE-TBS.

In this study, the wettability of NADES-TBS and AE-TBS particles was evaluated through contact angle and interfacial tension measurements, serving as an indicator of their Pickering emulsification capability. As shown in Figure 6, compared with the control group, 35.44 mN/m, NADES-TBS reduced the oil–water interfacial tension in the O/W, oil-in-water, model system to 28.13 mN/m, which was lower than that of AE-TBS, 30.91 mN/m. In the contact angle experiment, both NADES-TBS and AE-TBS showed contact angles greater than 90°, indicating that the particles were relatively hydrophobic and preferentially wetted by the oil phase. However, the contact angle of NADES-TBS (110.27°,110.08°), was closer to the theoretical optimum of approximately 90° for irreversible adsorption of particles at the oil–water interface than that of AE-TBS (132.17°,131.12°). This suggests that NADES-TBS had more balanced oil–water wettability, which may facilitate its adsorption and arrangement at the MCT-water interface. Together with its lower interfacial tension, these results support the stronger Pickering emulsifying performance of NADES-TBS compared with AE-TBS. (36)

### 3.8 Formation of Pickering emulsions

**Figure 7:**
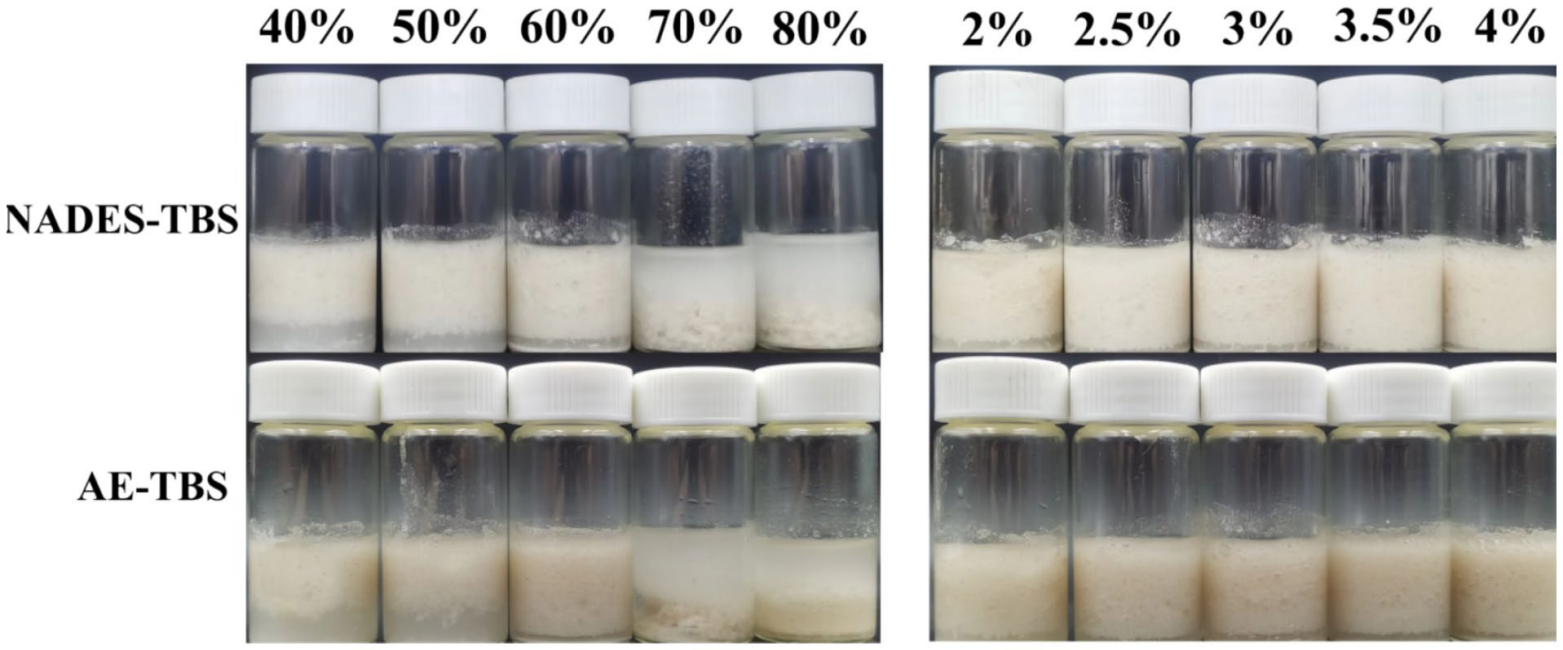
Effect of oil phase volume fraction and starch concentration on the formation of Pickering emulsions stabilized by NADES-TBS and AE-TBS.

Emulsifier concentration (*c*) and oil phase volume fraction (*φ*) are key factors influencing the formation of Pickering emulsions. Figure 7 shows the effects of oil phase volume fraction (40–80%) and emulsifier concentration (2–4%) on the stability of Pickering emulsions stabilized by NADES-TBS and AE-TBS. When *c* was fixed at 2%, increasing *φ* led to optimal emulsion stability at *φ* = 60% for both NADES-TBS and AE-TBS. When *φ* exceeded 60%, excessive oil was entrapped by starch particles, leading to phase separation or emulsion breakdown. At a fixed *φ* of 60%, emulsion stability rapidly improved with increasing *c*. No obvious phase separation was observed after 24 h for emulsions stabilized by NADES-TBS at *c* = 2.5%, whereas AE-TBS required *c* = 3.5% to show comparable short-term stability under the same observation conditions. These results indicate that NADES-TBS had better short-term emulsion-forming ability than AE-TBS. However, The appearance observations after 24 h indicate that NADES-TBS formed Pickering emulsions at lower starch concentrations than AE-TBS under the tested conditions. However, this 24 h observation should be considered a preliminary short-term stability assessment rather than evidence of long-term emulsion stability. Longer storage experiments, together with quantitative measurements such as creaming index, coalescence behavior, and droplet-size evolution, are needed to confirm storage stability. This observation is consistent with the results of interfacial tension and contact angle measurements (37).

### 3.9 Microstructure of Pickering emulsions

**Figure 8:**
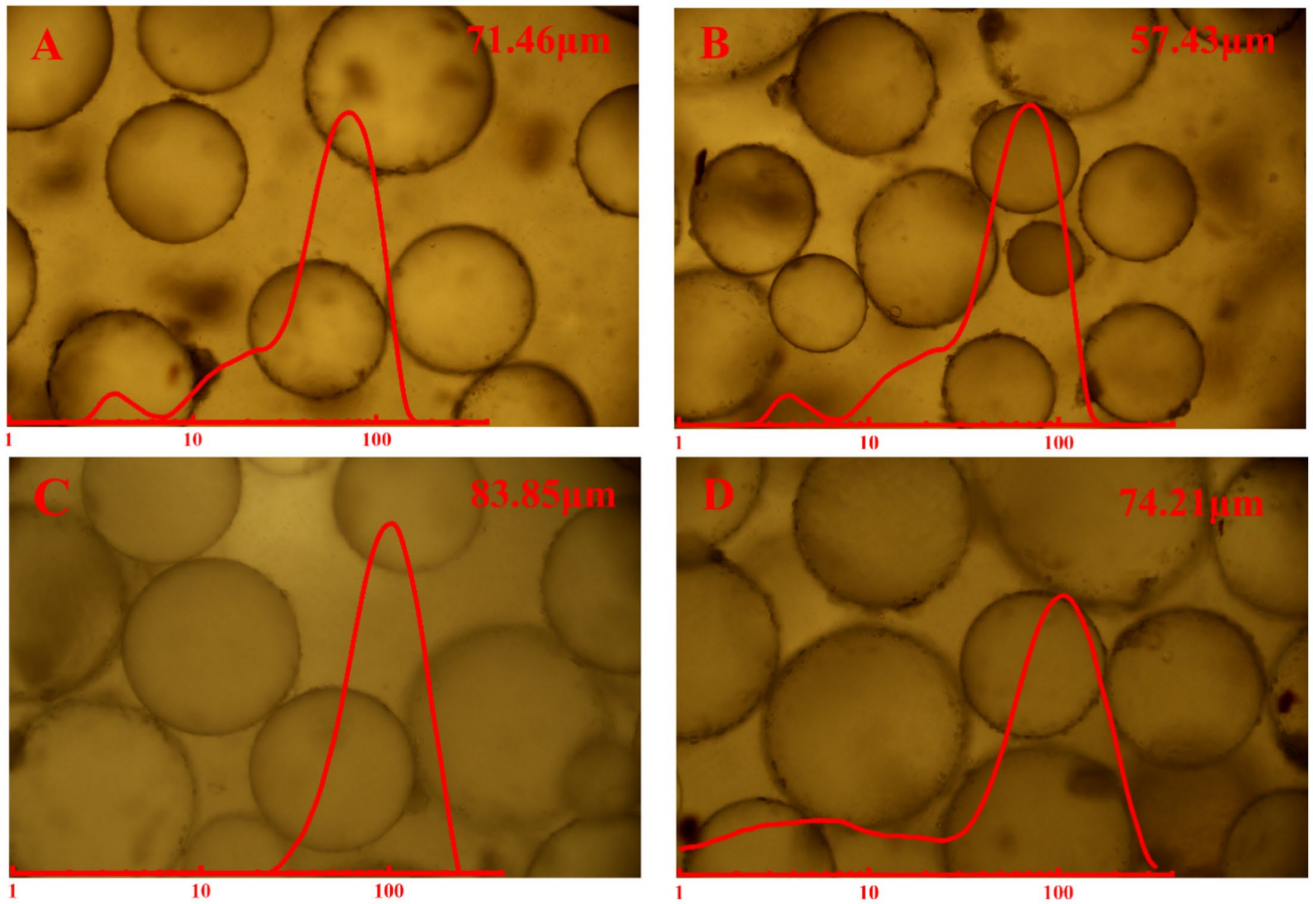
Microscopy images and oil droplet size distributions of medium-chain triglyceride, MCT-based, Pickering emulsions stabilized by NADES-TBS and AE-TBS. **(A)** Sample prepared with NADES-TBS at *c* = 3.5%, *φ* = 60%; **(B)** Sample prepared with NADES-TBS at *c* = 4.0%, *φ* = 60%; **(C)** Sample prepared with AE-TBS at *c* = 3.5%, *φ* = 60%; **(D)** Sample prepared with AE-TBS at *c* = 4.0%, *φ* = 60%.

The microscope images of medium-chain triglyceride, MCT-based, Pickering emulsions stabilized by NADES-TBS and AE-TBS under conditions of *φ* = 60% and *c* = 3.5% or 4.0% are shown in Figure 8. Under the applied homogenization conditions, both NADES-TBS and AE-TBS produced coarse MCT-based Pickering emulsions with droplet sizes in the micrometer range. At the same starch concentration and oil phase volume fraction, the droplets stabilized by NADES-TBS were smaller and more uniformly distributed than those stabilized by AE-TBS. However, the relatively large droplet sizes, 57.43–83.85 μm, indicate that the system should be regarded as a coarse Pickering emulsion rather than a fine emulsion. Further optimization of homogenization intensity, homogenization time, particle concentration, and particle surface properties may be required to obtain smaller droplets. The average droplet diameters of emulsions prepared with 3.5 and 4.0% NADES-TBS are 74.46 μm and 57.43 μm, respectively, while those stabilized by AE-TBS are 83.85 μm and 74.21 μm, respectively. A continuous and dense layer of particle adsorption is observed at the droplet interface, indicating that NADES-TBS effectively adsorbs at the oil–water interface and forms a stable particulate barrier. This is because increasing the emulsifier concentration with more starch particles meets the higher interfacial area demand, leading to reduced droplet size. Moreover, at the same concentration, emulsions stabilized by NADES-TBS exhibit smaller droplet sizes and a more compact structure compared to those stabilized by AE-TBS. (38) In Figure 8, MCT droplets are surrounded by starch particles, which are not only present at the oil/water interface but also dispersed in the aqueous phase. The starch particles in the water phase further enhance emulsion stability through a viscosity-enhancing effect.

### 3.10 EI and MVI of Pickering emulsions

**Figure 9:**
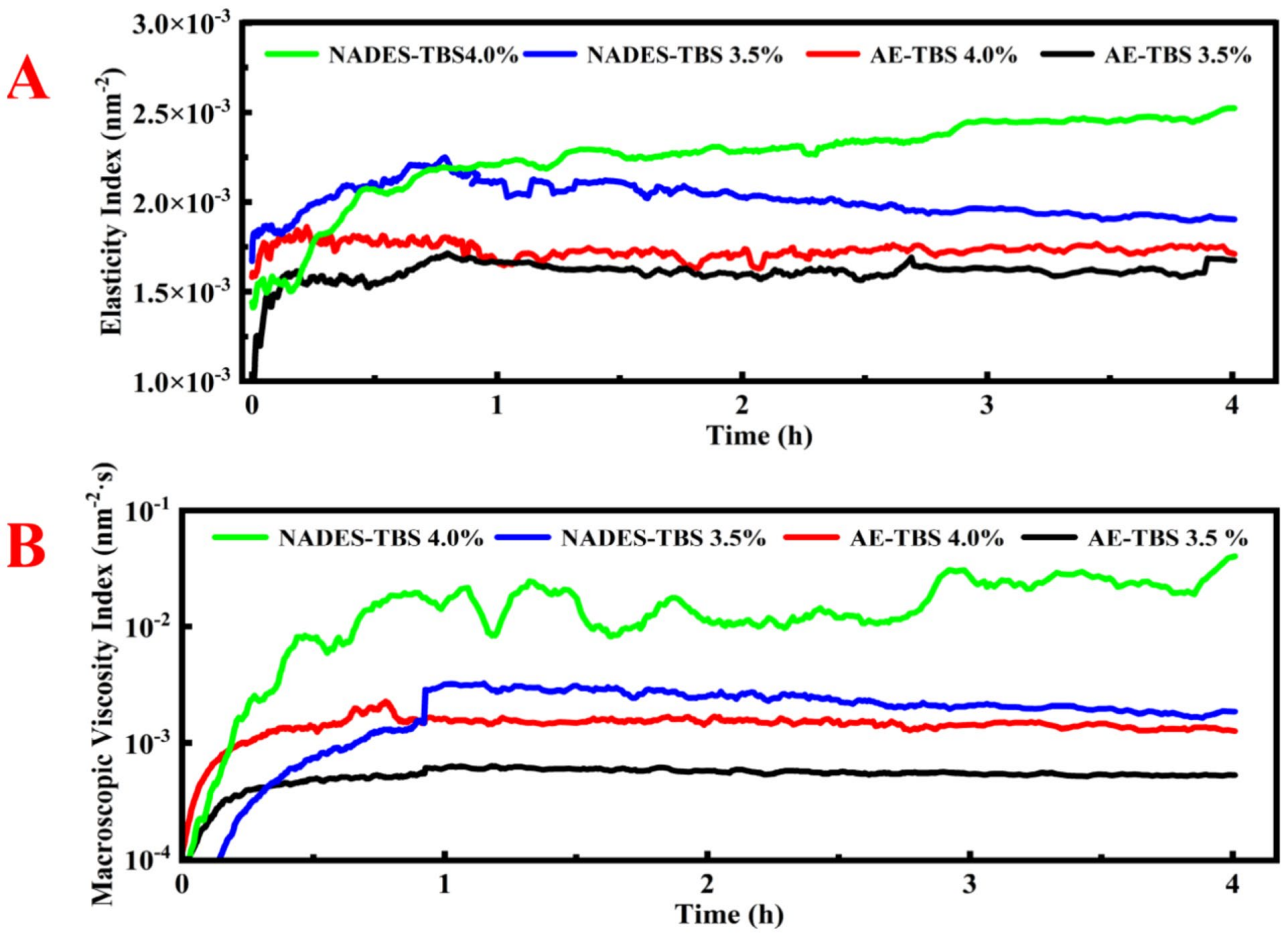
EI **(A)** and MVI **(B)** results of Pickering emulsions (*c* = 3.5 and 4.0%, *φ* = 60%) stabilized by NADES-TBS and AE-TBS.

Microrheology is a technique that uses diffusing wave spectroscopy (DWS) to study rheological properties, enabling the characterization of emulsion viscoelasticity without mechanical shear (39). The elasticity index (EI) represents the inverse of the distance between particles before network formation, corresponding to the strength of the system. The macroscopic viscosity index (MVI) reflects the sample’s viscosity at zero shear, which is closely related to the sample’s structure and rheological behavior. In this study, microrheology was employed to investigate the effect of Tartary buckwheat starch concentration on the EI and MVI values of emulsions. The EI and MVI profiles of emulsions stabilized by NADES-TBS and AE-TBS over 4 h are shown in Figure 9. As time progresses, both EI and MVI values increase and gradually stabilize after 3 h. With increasing concentrations of NADES-TBS and AE-TBS, both EI and MVI values rise consistently, indicating that the viscoelasticity and stability of the emulsion gel improve with higher starch loading. This is attributed to emulsion gels formed at higher starch concentrations having smaller droplet sizes and more compact structures. Pickering emulsions stabilized by NADES-TBS exhibit significantly higher EI and MVI values compared to those stabilized by AE-TBS, likely due to the smaller droplet diameter in NADES-TBS-stabilized emulsions, resulting in enhanced system stability and higher viscosity. These results demonstrate that NADES treatment enhances the emulsifying capacity of Tartary buckwheat starch, as evidenced by smaller emulsion droplet size, improved mechanical parameters, and superior emulsion stability (16). The improved short-term emulsion-forming behavior of NADES-TBS is likely associated with multiple observed factors, especially lower oil–water interfacial tension and a contact angle closer to 90°. Granule size and moderate aggregation may also contribute to interfacial coverage, but this mechanism requires further verification. The slight difference in relative crystallinity is unlikely to be the dominant factor. Importantly, the present control design does not allow all differences to be attributed solely to NADES, because AE-TBS and NADES-TBS underwent different extraction workflows. Native TBS, aqueous extraction controls, glycol-only and betaine-only controls, and process-equivalent controls should be included in future studies. In addition, because ethylene glycol was used in the present NADES system, quantitative residual solvent analysis by GC–MS or NMR, solvent recovery evaluation, and safety assessment are indispensable before any food-related application can be considered.

### 3.11 Practical implications and scalability considerations

From a practical perspective, the NADES method offers potential advantages in reducing the use of strong alkali and simplifying impurity removal. The possibility of reusing the NADES system also suggests potential economic and environmental benefits. However, several issues must be considered before industrial application. First, the relatively high viscosity of NADES may hinder mass transfer, increase mixing energy consumption, and complicate solid–liquid separation at large scale. Second, efficient solvent recovery and recycling are essential for economic feasibility. Third, because ethylene glycol was used in the present NADES system, residual solvent analysis and safety evaluation are required before food-related applications can be considered. Therefore, the present study should be regarded as a model-system evaluation, and further work is needed to assess solvent recovery, process cost, residual solvent levels, and scale-up performance.

## 4 Conclusion

In this study, a betaine–ethylene glycol NADES system was used as an alternative medium for extracting Tartary buckwheat starch, and the properties of NADES-TBS were compared with those of AE-TBS. NADES-TBS showed a lower apparent amylose content, 22.95%, than AE-TBS, 24.14%, while both samples retained the typical A-type crystalline structure. The relative crystallinity of NADES-TBS, 31.11%, was only slightly higher than that of AE-TBS, 30.39%, indicating that NADES extraction did not markedly alter the crystalline pattern. Compared with AE-TBS, NADES-TBS exhibited lower peak viscosity and setback viscosity, suggesting reduced paste viscosity and weaker starch chain reassociation during cooling. NADES-TBS also showed lower oil–water interfacial tension, 28.13 mN/m, than AE-TBS, 30.91 mN/m, and exhibited better short-term Pickering emulsion-forming ability under the tested conditions. These findings suggest that the selected NADES extraction workflow is associated with changes in granule morphology and selected physicochemical/interfacial properties of Tartary buckwheat starch. However, because residual solvent content, molecular-level structural changes, process-equivalent controls, and long-term emulsion stability were not fully evaluated, the present conclusions should be limited to the tested model system. Future work should include residual ethylene glycol analysis, FTIR, DSC, SEC/GPC, damaged starch content, amylopectin chain-length distribution, protein/lipid residue analysis, and long-term emulsion stability assessment.

## Data Availability

The original contributions presented in the study are included in the article/supplementary material, further inquiries can be directed to the corresponding author/s.
